# Awareness and knowledge of congenital cytomegalovirus infection among pregnant women and the general public: a web-based survey in Japan

**DOI:** 10.1186/s12199-021-01029-w

**Published:** 2021-12-21

**Authors:** Masayuki Kobayashi, Aya Okahashi, Kotoba Okuyama, Naomi Hiraishi, Ichiro Morioka

**Affiliations:** 1 Medical Affairs, MSD K.K., Kitanomaru Square, 1-13-12 Kudan-kita, Chiyoda-ku, Tokyo, 102-8667 Japan; 2 Department of Pediatrics and Child Health, Nihon University School of Medicine, 30-1 Oyaguchikamicho, Itabashi-ku, Tokyo, 173-8610 Japan

**Keywords:** Awareness, Congenital cytomegalovirus infection, Knowledge, General public, Pregnant women, Survey study

## Abstract

**Background:**

The best approach to reduce congenital cytomegalovirus infection (cCMVi) is to practice behaviors that reduce cytomegalovirus (CMV) transmission during pregnancy. Expanding awareness and knowledge of CMV is expected to result in increased practice of preventative behaviors. To this end, it is necessary to understand current awareness and knowledge of CMV.

**Methods:**

This web-based cross-sectional survey assessed the awareness and knowledge of cCMVi among pregnant women and the general public in Japan. Participants aged 20–45 years (pregnant and non-pregnant women, and men) were identified from a consumer panel. Study outcomes (all participants) included awareness of cCMVi and other congenital conditions. Among those aware of cCMVi, outcomes included knowledge of CMV transmission routes, long-term outcomes of cCMVi, and behaviors to prevent CMV transmission during pregnancy. Outcomes limited to pregnant women included the practice of preventative behaviors and opinion on how easy it is to implement these behaviors. The data of the pregnant group (pregnant at the time of the survey) were compared with those of the general group (non-pregnant women and men).

**Results:**

There were 535 participants in the pregnant group and 571 in the general group. Awareness of cCMVi was generally low (pregnant, 16.1%; general, 10.2%). Pregnant participants were significantly more aware of most congenital conditions than those in the general group, including cCMVi (*P* = 0.004). Knowledge about CMV/cCMVi was limited; there were no significant differences between the two groups for 24 of the 26 knowledge questions. A small proportion (one third or less) of pregnant women practiced behaviors to prevent the transmission of CMV, though most (73.3–95.3%) pregnant women who were aware of cCMVi considered such behaviors easy to implement.

**Conclusions:**

Awareness and knowledge of CMV/cCMVi is low among pregnant women in Japan; the level of knowledge is similar to that among the general public. This needs to be improved. Most pregnant women considered behaviors to prevent CMV transmission easy to perform, which indicates that effectively educating pregnant women regarding the long-term outcomes of cCMVi, CMV transmission routes, and preventative behaviors will contribute to a reduced incidence of cCMVi.

**Trial registration:**

UMIN Clinical Trials Registry, UMIN000041260.

## Background

Human cytomegalovirus (CMV) can cause serious disease when acquired congenitally or in immunocompromised individuals. Congenital CMV infection (cCMVi) occurs when CMV is transmitted from mother to fetus. The overall newborn prevalence of cCMVi is 0.64% worldwide [1], and in Japan, this prevalence is 0.26–0.50% [2–5]. Approximately 10–15% of infected infants show symptoms and 40–58% of symptomatic infants will develop sequelae [6–8]. Potential sequelae include hearing loss, vision impairment, intellectual disability, delay of psychomotor development, microcephaly, and cerebral palsy [9–11]. Between 10 and 21% of infected newborns who are asymptomatic at birth will develop permanent sequalae, of which sensorineural hearing loss is the most common [6, 12, 13]. There is currently no approved CMV vaccine, although its development is internationally prioritized [14].

Because there is no approved vaccine for CMV, the best strategy is to practice behaviors that can reduce the risk of maternal CMV infection during pregnancy. Infection occurs through direct contact with infected body fluids, such as saliva, urine, and semen [15]; thus, limiting the contact of pregnant women with the body fluids of others is important for prevention.

Although few studies on the awareness and knowledge of cCMVi have been conducted worldwide, existing studies indicate that there may be limited and variable awareness (12.5–60%) and knowledge of cCMVi and CMV-specific preventative behaviors among women [16–23]. Overseas studies have reported that even healthcare professionals do not have sufficient knowledge of cCMVi [24–27]. In Japan, two groups (a patient group of TOACH-no-Kai and a research group funded by the Agency for Medical Research and Development) have been working on raising awareness on mother-to-child infection through various methods, including developing and distributing leaflets on the subject. Efforts to raise awareness and knowledge about cCMVi are important because individuals without such awareness or knowledge, especially about serious long-term sequelae and specific behaviors that may prevent CMVi, are less likely to be motivated or able to effectively practice preventative behaviors during pregnancy and around young children.

Data regarding awareness and knowledge about cCMVi among pregnant women in Japan are scarce, and there are no data on this among the general public in Japan. However, to develop strategies to educate pregnant women and women who may become pregnant in the future, and to raise awareness of cCMVi and behaviors that prevent CMV infection, a thorough understanding of the current levels of cCMVi awareness and knowledge is needed. Two studies from Japan, one published nearly 10 years ago [16] and another published recently [18], reported the awareness of mother-to-child infections among pregnant women, including awareness of CMV in 18% and 33%, as well as their knowledge regarding transmission and methods to prevent maternal infection. Although both studies provided valuable information on these diseases and were conducted at Kobe University Hospital, which is renowned for its extensive research and education on mother-to-child infections, the single-center design of the survey studies represents a concern regarding generalizability. There has never been a survey of pregnant women in Japan to determine the level of implementation of behaviors associated with cCMVi prevention, the feasibility of those behaviors, or the level of knowledge regarding long-term outcomes for cCMVi. Such knowledge may contribute to increased practice of preventative behaviors [28]. Additionally, the authors speculate that a comparison of awareness and knowledge among pregnant women vs the general public (men and non-pregnant women) may provide insights into the effectiveness of current educational efforts among pregnant women. Therefore, a survey was conducted to determine the awareness and knowledge of cCMVi, including knowledge of long-term outcomes, and the practice of preventative behaviors among pregnant women broadly and the general public in Japan.

## Methods

### Participants

Potential participants were identified from a combined consumer panel consisting of 3900 pregnant women and 243,000 non-pregnant women and men in Japan aged 20–45 years. The panel was managed by INTAGE, Inc. (Tokyo, Japan) and Marketing Applications, Inc. (Tokyo, Japan). Survey invitations were sent to randomly selected potential participants from each target group.

Potential participants were eligible if they were aged 20–45 years (reflecting the approximate age range of Japanese women at delivery according to 2018 census data [29]), had no healthcare-related work experience or education, and had provided electronic informed consent. At the time of consent, the pregnant group comprised only women who were pregnant, and the general group comprised men and non-pregnant women (pregnancy status was self-reported).

### Study design

This was a web-based cross-sectional survey to assess the awareness and knowledge of cCMVi among pregnant women and the general public in Japan. The survey was implemented by INTAGE, Inc.

Potential participants received an email invitation to participate in the survey; the email contained a link to a website providing relevant study information and the option to provide electronic informed consent for participation. Those who provided electronic informed consent by checking “Agree” were asked to complete a screening questionnaire. If they met the inclusion criteria and did not meet the exclusion criteria, they could proceed to the main survey questionnaire. All participants were asked to answer questions about their demographic characteristics, including sex, age, education level, nationality, marital history, number of children, history of pregnancy, and gestational age (pregnant group only), and awareness of congenital conditions (i.e., whether they had heard of each disease), including cCMVi. Pregnant participants were also asked about their practice of preventative behaviors during pregnancy. Participants who answered that they had heard of cCMVi (herein referred to as “aware”) were asked additional questions regarding their knowledge related to CMV/cCMVi and whether they considered the practice of behaviors to prevent CMV transmission during pregnancy to be easy (pregnant group only). Those who answered that they had not heard of cCMVi (i.e., “unaware”) ended the survey. The questionnaire for the survey was newly developed based on prior publications primarily assessing cCMVi awareness, knowledge, and practices [16, 17, 19–27], and taking authors’ expertise and clinical experience into account.

Efforts to reduce non-response bias included restricting the total number of questions in the survey and the utilization of a two-step approach; in the first step, participants were surveyed regarding their awareness of cCMVi and other childhood diseases. In the second step, those who were unaware of cCMVi ended the survey at that point, while those who were aware of cCMVi were asked additional survey questions. Participants had to be within the approximate age range of women at the time of child delivery in Japan (20–45 years of age) to minimize bias from the disproportionate age distribution potentially present in online panels.

The study protocol was approved by the ethics review board of the Medical Corporation Toukeikai Kitamachi Clinic, and all participants provided electronic informed consent. The study was conducted in accordance with the Good Pharmacoepidemiology Practice [30] and applicable laws and regulations [31]. The study was registered at UMIN Clinical Trials Registry (UMIN000041260).

### Outcomes

Study outcomes included the awareness of cCMVi and other congenital conditions among all participants. Participants who had heard of cCMVi were asked about their knowledge regarding (1) CMV transmission routes, (2) long-term outcomes of cCMVi, and (3) behaviors to prevent CMV transmission (regardless of whether they practiced the behaviors). Outcomes limited to the pregnant group included the percentage of those who practiced behaviors related to the prevention of CMV transmission during pregnancy (included all pregnant women regardless of their awareness of cCMVi), and their opinion on whether it would be easy to implement these behaviors (included only pregnant women who were aware of cCMVi). Awareness of the existence of leaflets with information about mother-to-child transmitted diseases, sources of information for cCMVi awareness, and the experience of pregnant women regarding antibody screening for CMV were also determined.

### Statistical methods

This descriptive survey did not involve any confirmatory statistical tests; therefore, a power calculation was not performed. The target number of participants for each of the two groups was 500. Descriptive statistics were calculated for continuous variables; between-group comparisons were made using tests based on analysis of variance. Categorical variables were reported as number and percent of participants within each corresponding category; between-group comparisons were made using Fisher’s exact test. A *P *value of ≤0.05 was considered statistically significant. Analysis was performed using SAS Release 9.4 (SAS Institute, Inc., Cary, NC, USA).

## Results

### Participants

The survey questionnaire was distributed from July 2020 to September 2020. The background information of participants (total participants and participants who were aware of cCMVi) is shown in Table 1. Participants included 535 women in the pregnant group and 571 men and women in the general group. Over half of the participants were between 30 and 39 years of age (pregnant group, 60.6%; general group, 57.8%). Nearly all pregnant women (97.9%) and over half of those in the general group (54.8%) were either currently married or had been married previously. Most women who were not pregnant at the time of the survey had been pregnant previously (70.7%).

**Table 1:** Background information of questionnaire respondents

	Total		Participants who were aware of cCMVi	
	Pregnant group ***n =*** 535	General group ***n =*** 571	Pregnant group ***n =*** 86	General group ***n =*** 58
**Sex**				
Male	–	284 (49.7)	–	15 (25.9)
Female	535 (100)	287 (50.3)	86 (100)	43 (74.1)
**Age**				
Mean (SD)	31.5 (4.8)	31.8 (5.6)	32.2 (4.7)	31.4 (5.2)
20–29 years	184 (34.4)	195 (34.2)	24 (27.9)	20 (34.5)
30–39 years	324 (60.6)	330 (57.8)	56 (65.1)	34 (58.6)
40–45 years	27 (5.0)	46 (8.1)	6 (7.0)	4 (6.9)
**Education level**				
Junior high or high school	130 (24.3)	149 (26.1)	16 (18.6)	12 (20.7)
Junior college or vocational school	139 (26.0)	94 (16.5)	17 (19.8)	9 (15.5)
University	261 (48.8)	275 (48.2)	52 (60.5)	31 (53.4)
Graduate school or higher	5 (0.9)	45 (7.9)	1 (1.2)	6 (10.3)
No answer	0	8 (1.4)	0	0
**Japanese**	533 (99.6)	568 (99.5)	86 (100)	58 (100)
**Marital history**				
Have been married^a^	524 (97.9)	313 (54.8)	85 (98.8)	45 (77.6)
Never married	11 (2.1)	258 (45.2)	1 (1.2)	13 (22.4)
**Number of children**^**b**^				
0	277 (51.8)	306 (53.6)	48 (55.8)	15 (25.9)
1	186 (34.8)	119 (20.8)	29 (33.7)	20 (34.5)
≥2	72 (13.5)	146 (25.6)	9 (10.5)	23 (39.7)
**History of pregnancy**^**c**^				
Currently pregnant	535 (100)	–	86 (100)	–
Have been pregnant (not currently)	–	203 (70.7)	–	39 (90.7)
Never pregnant	–	84 (29.3)	–	4 (9.3)
**Gestational age**^**c**^				
1st trimester	19 (3.6)	–	2 (2.3)	–
2nd trimester	238 (44.5)	–	35 (40.7)	–
3rd trimester	278 (52.0)	–	49 (57.0)	–

Data are *n* (%)

^a^Either currently or previously

^b^Not including the fetus in the ongoing pregnancy

^c^Women only

*cCMVi* congenital cytomegalovirus infection, *SD* standard deviation

### Outcomes

**Fig. 1:**
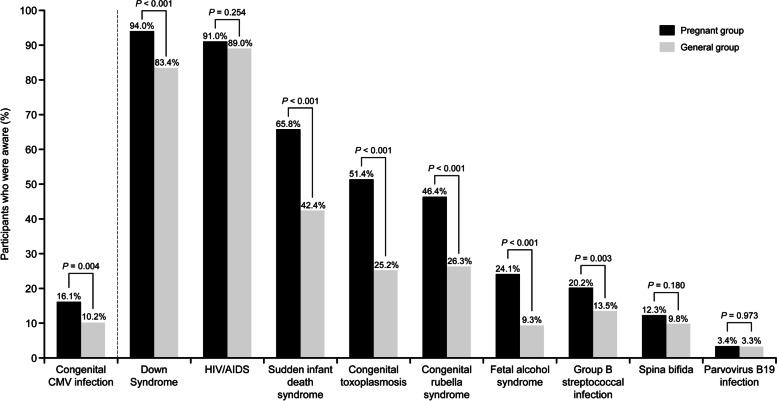
Awareness of congenital CMV infection and other congenital conditions. *AIDS* acquired immunodeficiency syndrome, *CMV* cytomegalovirus, *HIV* human immunodeficiency virus

Participant awareness of cCMVi and other congenital conditions is shown in Fig. 1. Pregnant women were significantly more aware of all congenital conditions, except for human immunodeficiency virus (HIV)/acquired immune deficiency syndrome (AIDS), spina bifida, and parvovirus B19 infection, than participants in the general group. While pregnant women were significantly more aware of cCMVi than participants in the general group (*P* = 0.004), awareness was generally low in both groups (16.1% [*n =* 86] and 10.2% [*n =* 58], respectively; 15.0% [*n* = 43] for non-pregnant women and 5.3% [*n* = 15] for men in the general group). The congenital conditions of which participants were most aware were Down syndrome/trisomy 21 (pregnant group, 94.0%; general group, 83.4%; *P* < 0.001) and HIV/AIDS (pregnant group, 91.0%; general group, 89.0%; *P* = 0.254). There was no substantial difference in the demographic distributions between the entire study population and those who were aware of cCMVi in the pregnant women group. Among those in the general group, women, those with a marital history, and those who had children comprised higher proportions of study participants who were aware of cCMVi (Table 1).

**Fig. 2:**
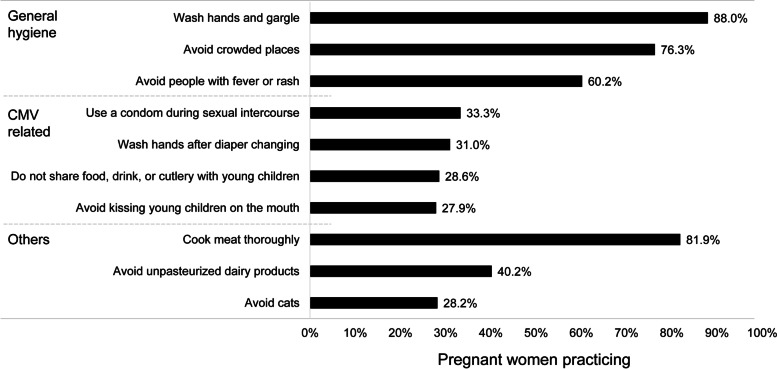
Percentage of pregnant women who practiced preventative behaviors during pregnancy (*n =* 535). *CMV* cytomegalovirus

In general, pregnant women practiced good hygiene, with most washing their hands and gargling, avoiding crowded places, avoiding people with a fever or rash, and cooking meat thoroughly (60.2–88.0%; Fig. 2). Fewer pregnant women implemented behaviors that can reduce the risk of CMV transmission, which include washing hands after diaper changing, avoiding kissing young children on the mouth, not sharing food, drink, or cutlery with young children (27.9–33.3%).

Knowledge of cCMVi (transmission route, long-term outcomes of cCMVi, and preventative behaviors) among participants who were aware of cCMVi was low overall for both groups, particularly regarding the long-term outcomes of cCMVi (Table 2). Most participants responded that they did not know what the long-term outcomes of cCMVi were (pregnant group, 62.8%; general group, 58.6%). The knowledge of behaviors that reduce the risk of CMV transmission was slightly higher than the knowledge of transmission route or long-term outcomes of cCMVi. Only around half of participants in each group knew that certain behaviors could reduce the risk of CMV transmission when interacting with small children (pregnant group vs general group: wash hands after diaper changing, 43.0% vs 48.3%; avoid kissing young children on the mouth, 46.5% vs 50.0%; not sharing food, drink, or cutlery with young children, 41.9% vs 55.2%). In the pregnant and general groups, 36.0% and 22.4% of participants, respectively, were unaware of any preventative behaviors.

**Table 2:** Knowledge on cCMVi among participants who were aware of cCMVi

	Pregnant group ***n =*** 86	General group ***n =*** 58	***P*** value^**a**^
**Potential transmission route**			
True			
Kissing	24 (27.9)	18 (31.0)	0.712
Changing diapers	25 (29.1)	10 (17.2)	0.117
Breast milk	9 (10.5)	14 (24.1)	0.037
Blood contact	18 (20.9)	20 (34.5)	0.084
Sexual intercourse	11 (12.8)	15 (25.9)	0.051
False			
Air conduction	6 (7.0)	5 (8.6)	0.756
Direct skin contact	11 (12.8)	8 (13.8)	1.000
Do not know	40 (46.5)	21 (36.2)	0.234
**Long-term outcomes of cCMVi**			
True			
Hearing loss	19 (22.1)	16 (27.6)	0.553
Developmental delay	14 (16.3)	12 (20.7)	0.515
Motor delay	12 (14.0)	9 (15.5)	0.813
Epilepsy	5 (5.8)	3 (5.2)	1.000
Visual problems	15 (17.4)	12 (20.7)	0.667
False			
Cardiac problems	14 (16.3)	4 (6.9)	0.125
Obesity	1 (1.2)	1 (1.7)	1.000
Increased risk for malignancy	2 (2.3)	3 (5.2)	0.392
Diabetes	1 (1.2)	3 (5.2)	0.303
Do not know	54 (62.8)	34 (58.6)	0.728
**Behaviors to reduce the risk of CMV infection**			
True			
Wash hands after diaper changing	37 (43.0)	28 (48.3)	0.609
Avoid kissing young children on the mouth	40 (46.5)	29 (50.0)	0.735
Do not share food, drink, or cutlery with young children	36 (41.9)	32 (55.2)	0.129
Use a condom during sexual intercourse	17 (19.8)	17 (29.3)	0.231
False			
Cook meat thoroughly	28 (32.6)	12 (20.7)	0.133
Avoid cats	28 (32.6)	12 (20.7)	0.133
Avoid unpasteurized dairy products	20 (23.3)	5 (8.6)	0.026
Do not know	31 (36.0)	13 (22.4)	0.098

Data are *n* (%) of subjects who selected each item as a correct answer

^a﻿^Calculated using Fisher’s exact test ^b^When interacting with young children

*CMV* cytomegalovirus, *cCMVi* congenital cytomegalovirus infection

**Fig. 3:**
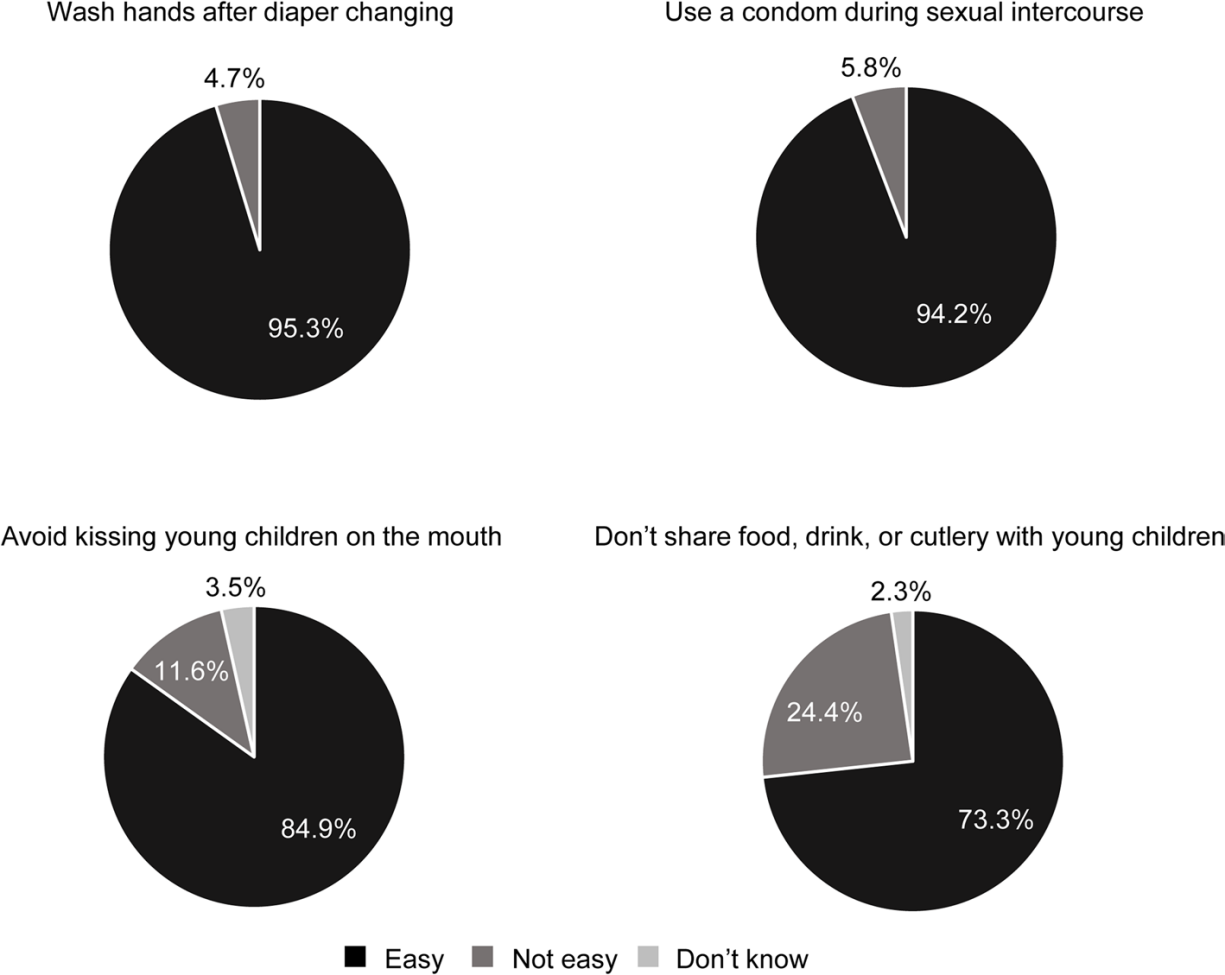
Opinion of whether preventative behaviors were easy to implement (*n =* 86). Pregnant women who were aware of cCMVi were included in this dataset. *cCMVi* congenital cytomegalovirus infection

Among pregnant women who were aware of cCMVi, 73.3–95.3% thought that behaviors that can prevent CMV transmission are easy to implement (Fig. 3). A small percentage of these women were aware of leaflets on mother-to-child infection created by a patient association or those by the Japan Agency for Medical Research and Development research group (7.0% each); 88.4% were unaware of either leaflet.

**Fig. 4:**
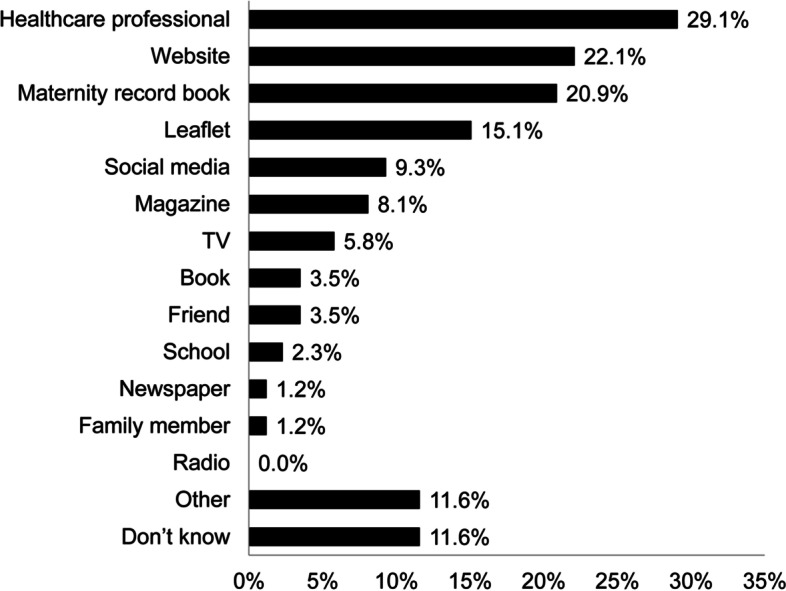
Source of cCMVi information among pregnant women who were aware of cCMVi (*n =* 86). *cCMVi* congenital cytomegalovirus infection

The information sources of cCMVi awareness among pregnant women who were aware of cCMVi are shown in Fig. 4. The most common source of information was healthcare professionals, who were cited by 29.1% of women. This was followed by websites (22.1%) and maternity record books (20.9%), which are shared between parents and healthcare professionals to monitor maternal healthcare throughout pregnancy.

Among pregnant women who were aware of cCMVi, 40.7% reported receiving information from a healthcare professional regarding antibody screening for CMV (current or past pregnancy), and 27.9% had antibody screening (current or past pregnancy).

## Discussion

Pregnant women (vs the general public) may be expected to be more aware and knowledgeable about cCMVi due to their interactions with healthcare providers and also heightened sensitivity towards information about diseases in children. In this study, we found low awareness of cCMVi among pregnant women (16.1%) and the general public (10.2%), and limited related knowledge among those who were aware of cCMVi. Considering the prevalence of cCMVi in newborns (globally, 0.64%; Japan, 0.26–0.50%) [1–5], the possibility of serious cCMVi-related sequelae [9–11], and the fact that there is no approved vaccine available, practicing behaviors that may reduce CMV transmission is the best available and effective method to reduce the risk of cCMVi [32]. Importantly, the level of cCMVi knowledge among pregnant women, for whom this information is most important, was not very high when compared with the level of knowledge among the general public, indicating a need for improved knowledge among pregnant women. A small percentage of pregnant women practiced behaviors that can prevent CMV transmission; however, pregnant women who were aware of cCMVi considered that such practices would be easy to implement.

Several surveys have reported rates of CMV awareness that were somewhat higher than we found in this study (pregnant group, 16.1%; general group, 10.2%); this is at least partly attributable to the populations surveyed. For example, awareness was 60% among pregnant women who were seen at two hospitals in France, including a teaching hospital with an active CMV prevention policy, and 52.5% among surveyed University of Milan attendees [17, 20]. A more recent study conducted at a single hospital in Rome reported an awareness rate of 59.1% [23]. Regarding these studies reporting relatively high awareness, it is important to note that the level of education is relatively high in some of the areas in which the participating institutions are located, and active education and screening on cCMVi are common. Lower rates of CMV awareness have been reported for studies conducted on more generalized populations in Japan (18%), the USA (20–22%), and the Netherlands (12.5%) [16, 19, 21, 22]. We found that current cCMVi awareness in pregnant Japanese women was similar to that reported by Morioka et al. nearly 10 years ago [16]. A more recent study conducted in Japan reported that 31.9% of pregnant women (non-healthcare professionals) were aware of CMV [18]. Although the latter study reported higher awareness, it should be noted that both Japanese studies were conducted at a single institution. Nonetheless, awareness remains inadequate. It seems that educational efforts for cCMVi have not substantially increased in the years between the studies.

Although pregnant women in the current study were generally practicing good hygiene (i.e., washing hands, gargling, avoiding crowded places, avoiding people with a fever or rash, and cooking meat thoroughly), most (66.7–72.1%) were not practicing behaviors that might specifically prevent CMV transmission (i.e., washing hands after diaper changing, avoiding kissing young children on the mouth, and not sharing food, drink, or cutlery with young children). Similarly, a study conducted in the Netherlands by Pereboom et al. reported that pregnant women had a high incidence of risk behavior [22]. Among women with children < 5 years old in their home, 91.3% reported sharing of utensils or cups with children, and 69.4% did not wash their hands after changing a diaper at least once during their pregnancy.

Overall, our study found that pregnant women in Japan had less knowledge about CMV transmission routes, long-term outcomes of cCMVi, and behaviors that reduce the risk of CMV transmission compared with similar survey studies conducted in other countries. It should be noted that, in some cases, the populations surveyed in the overseas studies differed somewhat from our study population. In our study, nearly half of the pregnant women who were aware of cCMVi had no knowledge about any of the routes of CMV transmission, and < 30% knew of each correct transmission route. Knowledge on CMV transmission was higher in the Italian study of university attendees. Among those who were aware of cCMVi, only ~ 15% had no knowledge about any of the routes by which CMV is transmitted, and approximately 40–70% were aware of each correct transmission route [20]. As noted above, however, the differences in study results may be attributable to the less generalized population surveyed in the Italian study.

Knowledge of the long-term outcomes of cCMVi was also low (1.2–22.1%). Only 22.1% of pregnant women in our survey were aware of hearing loss, while 42% (France, pregnant women), 48% (US, women), and > 50% (University of Milan attendees) of those surveyed outside of Japan were aware of this outcome [17, 19, 20].

Knowledge of preventative behaviors was reported for several of the overseas studies. Over 80% of the pregnant women surveyed in France [17] and approximately 50–75% of surveyed University of Milan attendees [20] were aware of at least one appropriate preventative behavior. Although this calculation was not done in our study, we instead found that 36.0% of pregnant women who were aware of cCMVi answered ‘Do not know’ when asked which listed behaviors could prevent CMV transmission, similar to the proportion in the general group (22.4%). Further, fewer than half of the pregnant women had known about each preventative behavior. Thus, our findings confirmed that preventative behaviors should be one topic of focus in the education of pregnant women.

Although knowledge and implementation of preventative behaviors were low, most pregnant women who were aware of cCMVi considered preventative behaviors to be easy to implement (73.3–95.3%). This suggests that the practice of preventative behaviors may increase dramatically if awareness and understanding of CMV transmission routes, preventative behaviors, and long-term outcomes of cCMVi are increased. Preventative behaviors would be particularly important for pregnant women who are in regular contact with infants, including those with older children who attend nursery school or kindergarten, those who work in a nursery school or kindergarten, and those who work in pediatric wards as health care providers. As such, increasing education and awareness of preventative behaviors among these women should be prioritized.

For the first time, the awareness among pregnant women of information leaflets on diseases transmitted from mother to child was reported and found to be quite limited. Low awareness about the leaflets among health care professionals may be one possible explanation for the low awareness among pregnant women, as healthcare providers are often a primary source of information for them [33]. The results of our survey highlight the need to increase activities to raise awareness among pregnant women. Efforts by healthcare providers to educate pregnant women should be enhanced and the daily activities of modern-day pregnant women should be carefully considered to help facilitate the development of better methods for dissemination of this information. Approaches that can target specific populations of pregnant women should also be explored.

The overarching finding of this study was that there is a need to improve awareness and knowledge of cCMVi among pregnant women in Japan. Towards this goal, healthcare professionals should stress the long-term outcomes of cCMVi and emphasize that they can be prevented by avoiding behaviors associated with CMV transmission during pregnancy. Additionally, more comprehensive strategies need to be evaluated, including providing incentives for the education of pregnant women and for antibody screening. As previously noted, the lifestyle and behavior of pregnant women should also be carefully considered when evaluating potential educational strategies. For example, mobile devices (i.e., handheld computers) are increasingly a part of daily life and may be considered as a potential platform to communicate information related to cCMVi.

This study has several limitations. First, the generalizability of the findings may be limited because online panelists may have different characteristics from the general population and also due to non-response bias. For example, the distribution of trimesters in pregnant women in our study, particularly the relatively low proportion of women in the first trimester compared with the proportion of women in the second or third trimester, may have introduced bias. The first trimester includes a period of time when the pregnancy is unconfirmed, and many pregnant women may be especially sensitive during the first trimester and, as such, may have refrained from responding to our survey, considering the topic. Second, the questionnaire was newly developed for the survey and was not validated; however, it was partially based on, and consistent with, several prior published studies [16, 17, 20, 25]. Therefore, the results should be interpreted with caution as this questionnaire had some limitations, including ambiguity in the knowledge questions (e.g., changing diapers does not necessarily constitute a direct risk of infection, whereas contact with infected urine is associated with an increased risk of infection). Finally, the results were subject to bias due to correct random guesses, which may have resulted in overestimation of awareness or knowledge.

## Conclusions

Awareness and knowledge of CMV/cCMVi among pregnant women in Japan were generally low and need to be improved, as both were similar to those among the general public, particularly in regard to cCMVi knowledge. Our survey revealed that many pregnant women considered behaviors that can prevent CMV transmission to be easy to perform; as such, effective education of pregnant women regarding CMV transmission routes, long-term outcomes of cCMVi and appropriate preventative behaviors will significantly contribute to reducing the burden of illness related to cCMVi.

## Data Availability

The dataset generated and analyzed in this study is not publicly available and cannot be shared with external researchers because consent on the dataset disclosure was not obtained from the participants.

## Abbreviations

AIDS: Acquired immune deficiency syndrome
cCMVi: Congenital cytomegalovirus infection
CMV: Cytomegalovirus
HIV: Human immunodeficiency virus
